# Universal Enzyme-Based Field Workflow for Rapid and Sensitive Quantification of Water Pathogens

**DOI:** 10.3390/microorganisms9112367

**Published:** 2021-11-16

**Authors:** Angela Sun, Jo-Ann L. Stanton, Peter L. Bergquist, Anwar Sunna

**Affiliations:** 1 Department of Molecular Sciences, Macquarie University, Sydney, NSW 2109, Australia; angela.sun@mq.edu.au (A.S.); peter.bergquist@mq.edu.au (P.L.B.); anwar.sunna@mq.edu.au (A.S.); 2 Department of Anatomy, School of Biomedical Sciences, University of Otago, Dunedin 9054, New Zealand; 3 Department of Molecular Medicine & Pathology, University of Auckland, Auckland 1142, New Zealand; 4 Biomolecular Discovery Research Centre, Macquarie University, Sydney, NSW 2109, Australia

**Keywords:** qPCR quantification, waterborne pathogen, enumeration, filtration, potable water, *Campylobacter jejuni*, *Cryptosporidium parvum*, *Giardia lamblia*, *Escherichia coli*, DNA extraction

## Abstract

A universal filtration and enzyme-based workflow has been established to allow for the rapid and sensitive quantification of leading pathogens *Cryptosporidium parvum, Giardia gamblia, Campylobacter jejuni,* and *Escherichia coli* from tap water samples with volumes up to 100 mL, and the potential to scale up to larger volumes. qPCR limits of quantification as low as four oocysts for *Cryptosporidium*, twelve cysts for *Giardia*, two cells for *C. jejuni*, and nineteen cells for *E. coli* per reaction were achieved. A polycarbonate filter-based sampling method coupled with the prepGEM enzyme-based DNA extraction system created a single-step transfer workflow that required as little as 20 min of incubation time and a 100 µL reaction mix. The quantification via qPCR was performed directly on the prepGEM extract, bypassing time-consuming, labour-intensive conventional culture-based methods. The tap water samples were shown to contain insoluble particles that inhibited detection by reducing the quantification efficiency of a representative pathogen (*C. jejuni*) to 30–60%. This sample inhibition was effectively removed by an on-filter treatment of 20% (*v*/*v*) phosphoric acid wash. Overall, the established workflow was able to achieve quantification efficiencies of 92% and higher for all four leading water pathogens, forming the basis of a rapid, portable, and low-cost solution to water monitoring.

## 1. Introduction

Waterborne pathogen monitoring plays a significant role in preventing and containing major public health problems worldwide. Despite advances in water treatment, sanitation, and hygiene, waterborne pathogen-related outbreak persists in all nations regardless of economic status. Notable bacterial waterborne pathogens include *Campylobacter* spp., a leading cause of bacterial diarrheal illness and a commonly identified cause of Guillan-Barré syndrome [1,2], and *Escherichia coli*, frequently associated with gastroenteritis and an indicator species for other faecal-borne microorganisms, such as *Salmonella* and *Hepatitis A* [3,4,5]. Protozoan parasites, such as *Cryptosporidium* and *Giardia,* are also prevalent in environmental water and are responsible for the majority of reported waterborne disease outbreaks due to protozoa worldwide [6]. These (oo)cysts-forming pathogens may cause disease in humans and farm animals and are often immune to water treatment due to their low infectious dose (1–25 oo(cysts)), resistance to chlorine and bromine treatment, and long viability periods of over 6 months [7,8,9,10].

The conventional methods for monitoring waterborne pathogens vary. These methods range from filtration- or gravity-based capture, immuno- or culture-based selection, direct counting, or immunoassays, many of which require specialised instrumentation confined to a laboratory setting [11,12,13]. These steps are often time-consuming, labour intensive, and costly. As early and reliable detection is crucial for disease prevention, a water screening method would ideally be field-deployable and provide high sensitivity, rapid turnaround time, require minimal infrastructure, and low cost. To this end, we have already demonstrated that a culture-independent prepGEM enzyme extraction system could be a time-saving alternative to conventional methods [14,15]. Proteinase prepGEM is a robust enzyme isolated from *Bacillus* spp. [16] that yields high quality, intact DNA from a wide range of bacteria, and can be used directly for PCR, qPCR, and NGS workflow [14,15,17,18] with as little as a 15 min digestion time. In this work, we aimed to integrate prepGEM DNA extraction into a rapid screening solution for field-settings.

Two microorganism capture strategies, filtration and affinity based, were considered for our rapid and economical prepGEM extraction workflow. When conjugated to a matrix or surface, antibody [19,20], or complement protein [21], capture methods can be highly effective at harvesting pathogens (>90%). These methods, however, are hard to scale up due to the cost of the antibodies and immune-separation particles [22], narrow target spectrum, and long elution or concentration time for the sample volume reduction [20]. Though initially trialled, this approach was quickly abandoned as not fit-for-purpose. In comparison, filter-based methods were favourable in terms of both cost and process time. At less than $1 USD each, filters are cheaper than antibodies and magnetic beads. Timewise, filters require no additional processing and are used directly in downstream processes. Filters are not without challenges: filter-based methods inadvertently concentrate inhibitors for downstream quantification and may be susceptible to blockage due to the presence of biofilms or particulates in the water samples. In this work, a filter-based workflow is developed for the prepGEM DNA extraction system and evaluated for robustness of capture and pathogen detection.

The pathogen load is generally quantified by standard culture-based methods coupled with manual counting [11,12,13,23], and these methods are the main contributors to extended reporting times. Flow cytometry has been proposed as an automated, high throughput alternative to manual counting where a recovery efficiency of over 90% has been achieved [24]. However, in-house studies with fluorescently labelled *Cryptosporidium, Giardia,* and *C. jejuni* (not shown) revealed mounting complexity to establish reliable enumeration algorithms due to auto-fluorescence, varying cell shape and size, life stages, and cell aggregation tendencies associated with environmental water matrices. These methods are not compatible with in-field deployment or low infrastructure cost. PCR-based detection methods, on the other hand, have been increasingly utilised against *Cryptosporidium*, *Campylobacter*, *Giardia,* and *E. coli* for their sensitivity, although many current reports remained qualitative (positive/negative) rather than quantitative, and the reported detection rates vary widely from 20% to 100% [25,26,27,28,29,30].

Here, we have developed a qPCR method that reliably quantifies water-borne pathogens at low concentrations. Together with the optimised filtration and prepGEM extraction processes developed in this work, the foundation is laid for a rapid field-based solution for pathogen monitoring in water.

## 2. Materials and Methods

### 2.1. Filter Capture and gDNA Extraction of Pathogens

Organisms used for the experiments were sourced as follows: *Campylobacter jejuni* (IFM 2454) and *Escherichia coli* O157 (IFM 2007) were obtained from IFM Quality Services Pty Ltd., Ingleburn, Australia. *Cryptosporidium parvum* (C10E7) and *Giardia lamblia* (G10E6) were purchased from Biopoint Pty Ltd., Sydney, Australia. For water sample spiking, live cultures of *C. jejuni* and *E. coli* were diluted to give approximately 300–1500 cells per PCR reaction, stock of *C. parvum* diluted to give 100–300 oocysts per PCR reaction, and stock *G. lamblia* diluted to give 50–100 cysts per PCR reaction.

Water samples (tap water and Milli-Q H_2_O) were spiked with pathogens prior to the filter capture process. Tap water samples were collected from the handwashing sink in Laboratory 6WW250 at Macquarie University (Sydney, Australia). Samples were collected across multiple days to provide sufficient variability. For sample volume of 100 mL or less, one Swinnex filter holder of 13 mm or 25 mm was used with filters of corresponding diameters. For sample volume of 500 mL, two of the 25 mm filters were used.

Extraction of gDNA was performed using the prepGEM Bacteria kit (MicroGEM, NZ) with a modified protocol detailed below. Up to two filters with captured cells were scrunched to fit tightly into the bottom of a 1.5 mL microcentrifuge tube, to which the extraction mixture was added (90 µL DNA-free water, 10 µL 10× GREEN+ Buffer, 1 µL prepGEM enzyme) followed by incubation in the thermocycler (75 °C-15 min; 95 °C-5 min). Extracted DNA was used immediately for quantification via qPCR, or stored at −20 °C.

Membrane filters were sourced from the following suppliers: hydrophilic white polycarbonate filter (0.2 µm-GTTP02500; 0.4 µm-HTTP02500; 0.6 µm-DTTP02500; 0.8 µm-ATTP02500, Merck-Millipore, Bayswater, Australia); hydrophilic brown polycarbonate filters (HTBP02500, Merck-Millipore); hydrophobic polytetrafluoroethylene (PTFE)–(WHA10411405, Sigma, Bayswater, Australia); Nitrocellulose filters (NC) (GSWP02500, Merck-Millipore).

### 2.2. Pathogen Enumeration

Direct quantification of *C. jejuni* cells was performed via 6 × 6 drop plate counting described by Chen et al. [31] and validated by the Australian Standard method for spread-plate enumeration (AS 5013.14.3-2012) [11].

To calculate the genome copy numbers of prepGEM-extracted gDNA from *C. jejuni* and *E. coli*, fluorometric assays were performed with the Qubit Fluorometer and Qubit dsDNA HS Assay Kit (Thermo Fisher Scientific, Scoresby, Australia) to determine concentrations of the gDNA extracted. The gDNA concentrations were then converted to genome copy numbers based on genome sizes [32,33] before being serially diluted to establish standard curves via qPCR.

Estimation of the *C. parvum* and *G. lamblia* oo(cysts) were derived from known concentrations from commercially prepared stocks (Biopoint, Sydney, Australia). The concentrations of gDNA extracted from the protozoan stock cultures were below the limit of detection for fluorometric methods; thus, Qubit assays were not performed.

### 2.3. Real Time Quantitative PCR (qPCR)

Primers for each pathogen (Supplementary Table S1) were screened for specificity via melt-curve analysis and amplification efficiency before the best performing assays were selected for further optimisation.

The qPCR reaction mix consisted of 30 µL qPCR of PowerUp SYBR Green Master Mix (Thermo Fisher Scientific), 6 µL each of Forward and Reverse primers of optimal concentration (Supplementary Table S2), and 18 µL of template taken either directly from the prepGEM extract or diluted as appropriate. This created a triplicate mixture of 20 µL each.

Real time PCR were performed on a LightCycler 480-II (Roche, Indianapolis, IN, USA) using the following thermocycling program: (1) UDG activation-50 °C, 2 min; (2) Dual-Lock DNA polymerase 95 °C, 2 min; (3) Quantification (x40 cycles)-Denature 95 °C, 15 s followed by Anneal/extend 60 °C, 1 m.

### 2.4. Optimisation of Primer Concentrations

Primers pairs were titrated in different final concentrations as shown in Supplementary Table S3. Combinations that resulted in higher qPCR efficiency (i.e., lower Cp values, Supplementary Table S2) were used for subsequent qPCR assays. For all primer screening and optimisation experiments, a no template control (NTC) that omits the target templates and a no amplification control (NAC) that omits the qPCR master mix were included.

### 2.5. Quantification and Statistical Analysis

Standard curves for qPCR-based quantifications were established using Cp values and the cell/genome copy numbers obtained through methods detailed in Section 3.2 regarding pathogen enumeration. In short, pathogen culture or stock of known concentrations were serially diluted 10-fold six times, and the diluted samples subjected to qPCR. The resultant Cp values were used in conjunction with the pathogen enumeration results to establish standard curves (Supplementary Table S4), which could then be used for qPCR-based quantification assays.

The quantification efficiency (%) of the workflow was determined by comparing the differences between spiked cell and estimated cell count based on Cp; Quantification efficiency % (QE) = Number of cells/genome copies detected from filter/Number of cell/genome copies spiked × 100%.

Limit of quantification (LoQ) was defined as the lowest concentration of pathogen that could be detected with qPCR in all three triplicates and yielded Cps with CV < 25%, where CV = Standard deviation (STD) of Cp/Mean Cp [34].

### 2.6. Water Sample Treatments

#### 2.6.1. Pre-Treatment, Post-Treatment, and Filter Treatments

The three treatment categories were summarised in the flowchart presented in Figure 2. Samples in the pre-treatment categories were treated prior to cell spiking to identify the effects of treatment on the sample matrix alone. Samples in the post-treatment categories were applied to spiked water samples to test the effect of treatment method on both the sample matrix and the cells. Samples in the filter treatment category were applied directly to the filter following the filter capture process to determine the treatment effect on the filtered sample matrix, cells, and the filter membranes.

#### 2.6.2. Activated Carbon Treatments

Granular activated coconut shell carbon, 12/40 mesh, (Pacific Water Technology, Seventeen Mile Rocks, Australia) was pre-washed gently in Milli-Q H_2_O three to five times to remove fine ash particles. Washed activated carbon was dried in a 70 °C oven before packed into a 10 mL syringe at 2 cm bed height for water sample treatment. Milk protein-coated activated carbon was prepared according to protocol described in Opet et al. [35] and dried before use.

#### 2.6.3. Zeolite, Silica Pellets, Silica Sand, and Chelex-100 Treatments

The zeolite, silica, and resin packing materials were sourced as below: treated zeolite 250–300 μm and 150–250 μm Avoca zeolite μm (Neptune Bio-innovations, Sydney, Australia); silica pellets 500 and 800 μm (Umang pharmatech, Maharashtra, India); silica molecular sieve type X13, 8 × 12 mesh (Fujian Anten Chemical Co. Ltd., Xiamen, China); Chelex-100 chelating resin (Bio-Rad, Gladesville, Australia); silica sand, acid-washed (Sigma-Aldrich, St. Louis, MI, USA).

The resins and minerals prewashed with Milli-Q H_2_O were packed into a syringe at 20% (*v*/*v*) of the water sample volume. The water sample was manually eluted through the packing materials at approximately 20 mL/min.

#### 2.6.4. Pre-Packed Silica Columns

Commercially available pre-packed columns (SEP-PAK C18, Alltech silica column, Alltech Diol column, and StrataX silica column) were used to pre-treat tap water samples prior to cell spiking. Water samples were eluted through the columns via gravity flow before being spiked with cells for capture and quantification.

#### 2.6.5. Filter Treatment with Phosphoric and Hydrochloric Acid

Acidic reagents (0.5 M hydrochloric acid, 1–25% *v*/*v* phosphoric acid) were used to wash the filter membrane. For sample volumes up to 10 mL, 3 mL of the acid wash was used to treat one filter. For sample volumes of 50, 100, and 500 mL, 10 mL of the acid wash was used per filter. If a rinsing step was included, the filters were rinsed with 3 mL Milli-Q H_2_O after the acid treatment.

## 3. Results and Discussion

### 3.1. Primer Screening and Optimisation

The primer sets (Table 1) were screened against the target template for their initial (non-optimised) specificity and amplification efficiency using a universal qPCR protocol described in the Materials and Methods section. The primers that generated high crossing point (Cp) or standard deviation of Cp (STD Cp) values and non-specific products were excluded from further analysis.

The selected primer sets (*C. jejuni*: HipO-F + HipO-R; *G. lamblia*: P241F + P241R; *C. parvum*: COWP P702F + COWP P702R; *E. coli*: uidA_F + uidA_R) were titrated for optimal concentrations (Supplementary Table S2). The determined optimal primer concentrations were used for the qPCR-based quantification analysis henceforth (HipO-F 250 nM + HipO-R 100 nM; COWP P702F 500 nM + COWP P702R 100 nM; P241F 500 nM + P241R 100 nM; and uidA_F 500 nM + uidA_R 250 nM).

The standard curves were established using serially diluted template DNA extracted using prepGEM of known genome copies (*C. jejuni* and *E. coli*) or cell numbers (*Giardia* and *Cryptosporidium*). The amplification efficiency of the qPCR was within 86–96%, and all displayed desirable R^2^ values (>0.98, Table 2) for all four pathogens. This result suggests that the primers were binding efficiently to the template, and the universal qPCR protocol worked well against different pathogen genomes. The high correlation value (R^2^) also highlights the efficiency of prepGEM enzymes in completely releasing the DNA material from all four species in as little as a 15 min digestion period. This is particularly impressive as protozoan (oo)cysts have tough outer protective layers that are a challenge for other extraction methods often requiring prolonged lysis steps or physical disruption, such as freeze-thawing or sonication, to release DNA [38].

### 3.2. Sensitivity of the qPCR Method for Pathogens Quantification

Table 2 gives both the measured analytical limit of quantification (LoQ) and the limit of detection (LoD) for each qPCR assay against the four pathogen genomes. The LoQ for *C. jejuni* was two genome copies, which compared favourably to other molecular-based quantification techniques, including a qPCR quantification method for poultry samples achieving an LoQ of 31 copies per qPCR reaction [40], and a filter-based qPCR method with the LoQ ranging from 10 to 100 *C. jejuni* cells per 100 mL of filtered sample [41]. Moreover, the latter required cycles of freeze-thaw lysis add to the processing time and may be difficult to perform in the field.

For *Cryptosporidium* and *Giardia*, our qPCR setup achieved an LoQ as low as four oocysts and 12 cysts per PCR reaction, respectively, which compared favourably to other molecular-based methods typically reported for protozoans [38].

The PCR-based detection limits reported in the literature for *Cryptosporidium* range widely from 1 to 10^6^ oocysts [42], with the majority detecting between 10 to 100 oocysts [43]. These methods all required immunomagnetic separation and/or centrifugation for enrichment, which are expensive and cumbersome when translated to a larger scale [44,45]. The molecular based detection limit for *Giardia* has been reported in the literature to similarly range from 10 to 100 cysts with commercial kits (ViPrimePLUS *Giardia* intestinalis qPCR Kit, Vivantis).

Comparable to our LoQ, a qPCR-based detection level as low as a single cyst of *G. lamblia* and one oocyst of *C. parvum* has been reported previously [38]. The workflow published by Inglis and Kalischuk [32], however, required a 1 h incubation with a lysis buffer in addition to freeze-thaw cycles and sonication for optimal DNA yield (~83% recovery), followed by qPCR incorporating a Taqman probe. In comparison, our workflow requires only a two-step incubation with prepGEM, followed by a primer-only qPCR, thus offering a more economical and streamlined approach suitable for potential field deployment.

The LoQ for *E. coli* was 19 genome copies per reaction (Table 2), slightly higher than the <10 copies LoQ reported by Walker et al. [29], from whom the primers against *E. coli* for this work were based, and by Shretha et al., where <10 copies LoQ could be achieved using a USEPA-qPCR method [30]. Although there are conventional methods, such as Colilert-18, that also provide simple and sensitive monitoring against *E. coli* (1 organism/100 mL), an incubation step is often required. Another highly sensitive molecular-culture hybrid method, MPN-qPCR, has been reported to achieve an LoQ of 1 CFU/g in a vegetable matrix but requires overnight cultivation [46], so it is not possible to deploy in the field.

In summary, the qPCR workflow described here permits the quantification with fast turnaround of equal or greater analytical sensitivity (LoQ) compared to culture-based or other molecular-based methods. The low limits of detection (LoD) shown here for these pathogens may help in water monitoring where the acceptable legal microorganism limits are extremely low, for example, one cell of *E. coli* per 100 mL, and one *Cryptosporidium* and *Giardia* oo(cysts) per 10 litres of water [47,48].

### 3.3. Primer Performance in a Mixed Pathogen Community

The performance of primers in the presence of other DNA templates was evaluated in a mixed-pathogen sample to mimic the complex nature of environmental water. In silico analysis had shown these primers to be specific, but a possibility remains that a mixed microbial and aquatic community may affect the sensitivity and specificity of the qPCR assays.

To evaluate qPCR assay performance in a mixed species community, we compared the relative quantification efficiencies (rQE) between single and contrived pathogen mixtures containing the same number of each pathogen (Table 3), where relative quantification efficiency (rQE%) = QE in mixed-pathogen sample/QE of single-pathogen sample × 100%.

At a small scale (10 mL), the rQE% ranged between 82% and 119% in a mixed pathogen sample. The efficiency values fluctuated and sometimes exceeded 100% due to variations in Cp and the low cell count (20–24 cells) per reaction. Similar works describing qPCR-based detection methods reported primer specificity in a single-pathogen sample [49,50] within the same range, but the qPCR assay performance in a mixed-pathogen environment was often not quantified. The results from this experiment suggest that the ability to target specific pathogens could be influenced but not significantly compromised by the presence of other microorganisms.

### 3.4. Selection of Suitable Filter for Pathogen Capture

**Figure 1:**
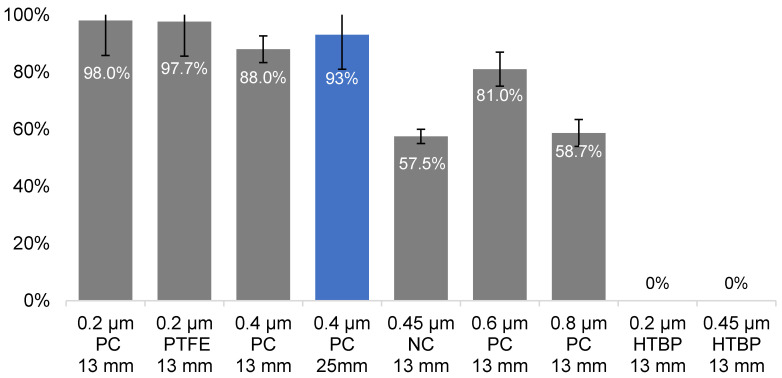
Capture efficiency of *C. jejuni* cells with various filter types. Pore sizes: 0.2–0.8 µm; filter types: PC—hydrophilic polycarbonate filter, PTFE—hydrophobic polytetrafluoroethylene, NC—nitrocellulose, HTBP—hydrophilic polycarbonate filter-brown; filter diameters—13- or 25-mm. Blue bar—filter type selected for further experimentation.

Filters of pore sizes ranging from 0.2 to 0.8 µm and diameters of 13 mm or 25 mm of various materials were tested. Materials tested were polycarbonate (PC), brown polycarbonate (HTBP), polytetrafluoroethylene (PTFE), and nitrocellulose (NC) for suitability for environmental water sampling. *C. jejuni* was used as a target pathogen as it is the smallest amongst the four organisms, thus setting the minimal pore size requirement. A slight loss of cells was associated with pore sizes over 0.2 µm, but, at 0.4 µm, most of the cells could be captured using either the 13 or 25 mm filters (Figure 1, 88% and 93%, respectively). While smaller pore size helped increase the pathogen capture rate, it was also associated with drawbacks, such as slower flowrate, higher back pressure, and a lower total filtration volume, due to its predisposition to block. Water pre-filtration or pre-treatment may be a solution to blockage but may lead to cell loss of 20–80%, as reported below and elsewhere [51], and should, therefore, be avoided unless dealing with highly turbid samples.

The water flow rate through the 0.4 µm filters was 18 mL/min × cm^2^/psi (Isopore membrane filter, HTTP01300/02500, Merk-Millipore), almost six times higher compared to that of the 0.2 µm filters (3.36 mL/min × cm^2^/psi, GTTP01300/02500, Merk-Millipore). With the high capture efficiency and faster flow, the 0.4 µm filters were best suited for our workflow as they could retain most of the *C. jeuni* cells while reducing the sample processing time. Additionally, the 0.4 µm Isopore filters were thinner (10 µm) compared to the other pore size varieties (25 µm for 0.2, 0.6, and 0.8 µm). This meant that the 0.4 µm filters could compact to smaller volumes requiring less prepGEM extraction reagent for DNA extraction. This allowed the processing of DNA captured from up to 500 mL samples (two of 25 mm filters) in one prepGEM reaction, thus reducing the overall cost.

In terms of the filter materials, polycarbonate (PC) membrane is a typical choice for capturing *Campylobacter* sp., followed by nitrocellulose (NC) membranes [52]. A brown variety of the PC membrane (HTBP) was also tested. The polytetrafluoroethylene (PTFE) was included since *Campylobacter* sp. were found to have a slightly hydrophobic surface [53] and, thus, may adhere better to hydrophobic membranes.

The PC membranes were found to capture better compared to the NC filters (Figure 1, 88–98% vs. 57.5%, respectively). While the performance of the PTFE filters was comparable to the PC filters, they were not as convenient due to the pre-wetting requirement. The HTBP filters were found to be incompatible with the prepGEM extraction process as the brown colouration dissolved into the reaction during the 72 °C digestion and seemed to interfere with the subsequent qPCR. Based on the results shown in Figure 1, the PC-based filters were best suited for our workflow.

### 3.5. Treatment Strategies for Tap Water Matrix

Contaminated potable water, such as tap water and low turbidity environmental waters from wells, ponds, or streams, is one of the main means of transmission for *Cryptosporidium* and *Giardia* (oo)cysts, and a common reservoir for *Campylobacter* and *E. coli*. In this work, tap water was used to represent a low turbidity potable water matrix for method development.

**Figure 2:**
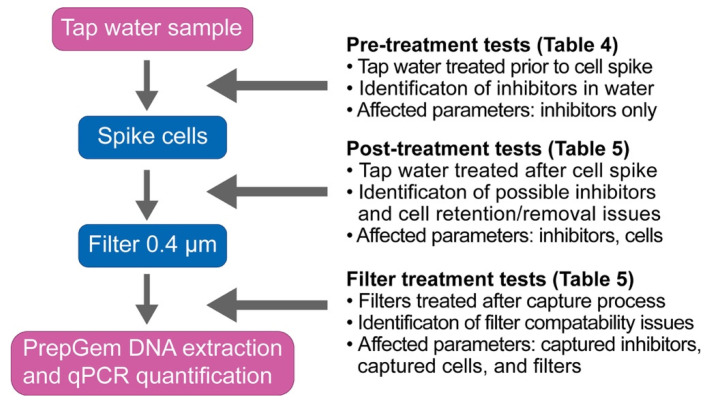
Experimental setup for the three treatment tests against tap water samples: pre-treatment, post-treatment, and filter treatment. Arrows indicate the timing of the application of the corresponding treatments.

The treatment strategies were applied at different points of quantification workflow, as illustrated in Figure 2 (pre-treatment, post-treatment, and filter treatment), to identify the effects on the cells, water matrix, and filters separately.

Untreated tap water samples spiked with *C. jejuni* cells produced a relative quantification efficiency of 40–60%, fluctuating daily (Table 4). This indicates the presence of inhibitory agents in the tap water against the prepGEM, qPCR assay, or both. Common substances found in tap water include inorganic metals, chlorine, and fluorine from water treatment, limescales, such as CaCO_3_ and MgCO_3_, and other suspended particulates from the delivery system. The effectiveness of the pre-filtration (PF-TW, 60–80%) and centrifugation (G-TW, ~100%) suggested that some inhibitors may be in the form of suspended particulate.

The preliminary water analysis suggests the presence of trace amounts of metals and some CaCO_3_ solids (Supplementary Table S5). These substances were targeted in our treatment strategies using a chelating agent, such as Chelex-100, silica pellets, or pre-packed silica columns (Alltech, StrataX, SEP-PAK C18), to remove the metals and fine particulates in the water (Table 4). Treatment with all-purpose water purification agents, such as zeolite or activated carbon with and without a milk protein coating that prevents the adsorption of bacteria, were also explored. The good performance from some zeolite (ZT-TW, 79%), activated carbon (AC/MP-TW, 103%), and silica-based methods (Si5.2-TW, 100%) may be owing to their ability to remove metal ions or particulates, although conclusions cannot be made without further testing.

### 3.6. Assessing Compatibility between the Water Treatment and Filtration Processes

While some pre-treatment methods were found effective at removing the inhibitors from tap water prior to the spiking with the pathogen, the compatibility with downstream capture and quantification processes must also be considered. For example, resin or column-based treatment may remove cells from water samples, preventing accurate downstream quantification. To test the compatibility between the water treatment and filter-capture, the best performing treatment methods based on the pre-treatment results (zeolite, silica pellets 500 µm, activated carbon with milk coating, and the Chelex-100 treatment) were used in a post-treatment setting (Figure 2, post-treatment tests); the results are shown in Table 5. Despite promising results during pre-treatment testing, the QEs from the post-treatment strategies were either low (<60% and lower for zeolite-TWC-ZL, activated carbon with milk protein-TWC-AC/MP, and Chelex-TWC-CX treatments, Table 5) or highly variable (30–100%, silica pellets-TWC-Si5.2, Table 5). Considering the significant drop of the QE between the pre- and post-spike samples, it was speculated that the *C. jejuni* cells may be retained by the highly porous activated carbon and zeolite matrix or caught in the tightly packed Chelex and silica resins, preventing accurate enumeration.

### 3.7. Acid-Wash Filter Treatment

As the options for post-treatment were limited due to the cell retention, we explored an alternative process where only the filters were treated following the pathogen capture (Figure 2). The main disadvantage for direct filter treatment is the possible corrosion and/or destruction of the cells or filters if treated with harsh reagents.

An acid treatment using 0.5 M HCl was considered based on the earlier observation (Table 4) that inhibitors were fine particulates, and possibly insoluble inorganic salts, such as CaCO_3_ and MgCO_3_. This hypothesis was confirmed by the successful removal of the inhibitor after acid treatment (QE >96%, TWC-HCl, Table 5). Furthermore, a high QE after the acid treatment implied that the cell and filter integrity were not significantly compromised. At a 10 mL scale, this was a proof of concept that acid treatment was an effective method to remove inhibitors from tap water samples. 

### 3.8. Upscaling Pathogen Capture Workflow—50 mL to 500 mL Water Volume

Following the effective acid-wash filter treatment at 10 mL, strategies were explored to scale up the treatment to larger water volumes more typical of the sample volumes used for environmental water testing.

The main challenge associated with scaling up a filter capture method is the removal of inhibitors, which also increase due to the larger sample volume that needs to be processed. The inhibitor elimination may require a combination of the following adjustments: (1) higher volume acid wash, (2) use of a stronger acid, and (3) use of more concentrated acids under conditions that do not corrode or destroy the polycarbonate filters and cells.

**Figure 3:**
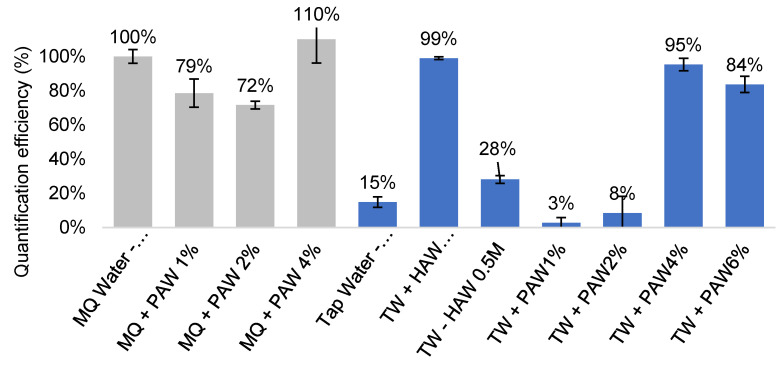
Effectiveness of inhibitor removal after various acid treatment in 50 mL filter-captured *C. jejuni* samples. PAW—phosphoric acid wash, filters treated with 10 mL of 1–6% phosphoric acid after capture. HAW—hydrochloric acid wash, filters treated with 10 mL of 0.5 M hydrochloric acid after capture. Grey columns—control samples with Milli-Q water (MQ) to assess effect of acid treatment on filters alone. Blue columns—quantification performed in spiked tap water (TW) samples. S—10 mL sample volume. All other samples were in 50 mL volumes.

Although 0.5 M HCl was found to be an effective treatment, it could potentially cause minor corrosion at a higher concentration (2.4 M HCl or 20% *v/v*, [61]) and hydrolyses cells. Phosphoric acid, a weaker acid that does not corrode PC filters at high concentration (>40% *v/v*, [61]), was thus trialled at 1–6% (*v/v*) alongside 0.5 M hydrochloric acid at a 50 mL scale (Figure 3). A phosphoric acid wash (PAW) treatment at 4% and 6% was found to be effective at removing the inhibitors from 50 mL tap water samples (TW + PAW4%-95% QE; TW + PAW6%, 84% QE, respectively, Figure 3) without compromising the filters. In comparison, the filters treated with 0.5 M HCl resulted in a significant drop in the QE from the 10 mL sample volume with 3 mL acid wash (TWC + HAW 0.5 M-S, 99% QE, Figure 3) to 50 mL sample volume with 10 mL acid wash (TWC + HAW 0.5 M, 28% QE, Figure 3), possibly due to filter corrosion or cell hydrolysis. We proceeded with PAW as it has a milder effect on the filters and cells. This was further demonstrated using MilliQ-H_2_O, showing a minimal effect on the QE when phosphoric acid wash was applied (Figure 3). Being a weak acid, phosphoric acid was also considered safer to handle than hydrochloric acid in a field setting.

**Figure 4:**
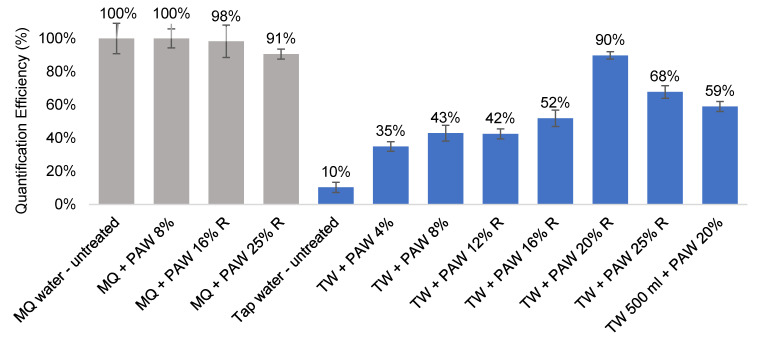
Effectiveness of inhibitor removal after phosphoric acid wash (PAW) treatment in 100–500 mL filter-captured *C. jejuni* samples. PAW—phosphoric acid wash, filters treated with 10 mL of 8–25% (*v*/*v*) phosphoric acid after capture. -R suffixes represented an additional rinsing step with 3 mL Milli-Q H_2_O post-wash. Grey columns—control samples with Milli-Q water (MQ) to assess effect of acid treatment on filters. Blue columns—quantification performed in spiked tap water (TW) samples of 100 mL volume, except otherwise labelled (500 mL).

Proportionally more phosphoric acid is required to remove the inhibitors from larger volumes. This could be achieved either by a larger sample volume or higher acid concentrations. Considering that portability is an important factor for field deployment, it was decided that using a higher acid concentration while keeping the wash volume low (10 mL) would be more desirable. This was tested using 100 mL water samples. Phosphoric acid washes of increasing concentration (4%, 8%, 12%, 16%, 20%, and 25%) were used to treat the pathogen capture filters, and the optimal QE was achieved at 20% (*v/v*) phosphoric acid (TW + PAW 20% R–90% QE, Figure 4). At this concentration, the filters and cells were not compromised by the acid treatment, as shown by the MilliQ-H_2_O control samples (Grey columns, Figure 4). It was, however, necessary to add a rinsing step with 3 mL Milli-Q-H_2_O post acid treatment for the acid washes higher than 12% to completely remove the residual acid reagent (data not shown).

The efficacy of the acid treatment was tested on a 500 mL sample volume. With 10 mL of 20% *v*/*v* phosphoric acid wash, a QE of 59% could be achieved. At this volume, filter blockage became an issue, and two polycarbonate filters of 0.4 µm pore size and 25 mm diameter were required. However, no extra prepGEM reagent was needed as the polycarbonate filters were thin enough to fit two into a single 100 µL reaction volume. The thickness of the filters played a role in cost reduction and should be sourced at 10 µm or thinner, if possible, to minimise the prepGEM volume required for DNA extraction.

In conclusion, an acid wash of 10 mL, 20% (*v*/*v*) phosphoric acid effectively removed the insoluble particles, most likely insoluble salts, such as CaCO_3_ and MgCO_3_, from the tap water samples. As a result, a quantification efficiency of 95% in 50 mL and 90% in 100 mL could be achieved with the acid treatment (Figure 3 and Figure 4), a significant improvement from the untreated tap water samples (QE 30–60%, Table 5). The potential for larger volume testing has been demonstrated with two PC filters instead of one, and a QE of 59% was achieved. The further optimisation of large volume sample treatment, such as larger acid wash volume or higher acid concentration, may help improve the QE.

### 3.9. Multi-Pathogen Quantification in a 100 mL Tap Water Sample after Phosphoric Acid Treatment

Our qPCR/prepGEM/acid wash pathogen capture system resulted in a pathogen detection efficiency of between 87% and 100% (Table 6) from a 100 mL mixed pathogen tap water matrix. This is in line with, or higher than, many similar filter-based methods (Supplementary Table S6).

A significant hurdle to high quantification efficiency from water samples is cell loss during the recovery process. Filtration alone may lead to significant cell loss, as suggested by Hu et al. [51]. Other forms of recovery, such as microfluidics (Ishii et al. [62]) and flow cytometry (Keserue et al. [63]), also identified cell recovery as a challenge to accurate quantification. However, with appropriate adaptations, filter-based capture has the potential to achieve high recovery, as demonstrated in this work (>87%) and others (70% and 54.9%, Al-Sabi et al., [64]).

The extraction of DNA from filtrated cells must also be robust and efficient to ensure high quality DNA templates and the authentic representation of all the pathogens in the sample. For field deployment, this also must be achieved quickly. This is sometimes difficult due to the inhibitory effects of the matrix (Guy et al., Ishii et al. and Papić et al. [38,40,62]), or it requires extra incubation time and non-portable instruments, such as high speed centrifuges (Rudi et al., [65]). We have demonstrated that the prepGEM mix was able to satisfy both requirements as it could effectively release DNA from pathogens in 20 min after a simple and field-friendly one-step acid treatment of the capture filter to remove inhibitors.

The sensitivity and efficiency of the qPCR assays used here was on par with other qPCR work, such as Ishii et al. [62]), and as discussed in Keserue et al. [63]. The multi-pathogen qPCR results in Table 6 further demonstrate that the qPCR efficiency has not been negatively affected in larger scale samples (87–100% at 100 mL sample volume) when compared to our smaller-scale results at a 10 mL sample volume (89–119%, Table 3). The rapidity and scalability of a filter capture-prepGEM-qPCR workflow would be a distinct advantage for field deployment.

### 3.10. Final Workflow for Multi-Pathogen Quantification in Tap Water

A universal prepGEM enzyme-based workflow has been established to allow for the rapid and sensitive quantification of leading pathogens from environmental waters. The general protocol is described below:

1. **Filtration**—water samples up to 100 mL filtered through one 25 mm Swinnex adapter with a polycarbonate filter (10 µm thickness, 25 mm diameter, 0.4 µm pore size, HTTP02500, Merck-Millipore, AU) using a syringe or pump. Two filters may be required for sample volume up to 500 mL if blockage occurs.
2. **Treatment**—to remove inhibiting particulates in tap water (or similar matrix), the filter was treated with 10 mL of 20% (*v*/*v*) phosphoric acid before eluting using a syringe or pump, followed by rinsing with 3 mL of MilliQ-H_2_O.
3. **DNA extraction**—filters were removed carefully from the Swinnex adapter and folded and squashed to fit into the bottom of an Eppendorf tube so it could be totally submerged in the 100 µL prepGEM reaction mix. Care must be taken to not touch the side with the filtrate. The mixture was then incubated at 75 °C for 15 min for digestion, and 95 °C for 5 min for enzyme inactivation.
4. **Quantification**—quantification via qPCR could be performed immediately following the DNA extraction without further treatment. Thermocycling and subsequent quantitative analysis performed as detailed in the Methods section.

## Figures and Tables

**Table 1:** Evaluation of the efficiency of primer sets targeting *C. jejuni*, *G. lamblia*, *C. parvum*, and *E. coli* at 500 nm final primer concentration. Lower MeanCp suggests better amplification efficiency. *—Primer sets selected for further optimisation.

Primer Set	Target	MeanCp	STD Cp	Comments	References
HipO-F + HipO-R *	*C. jejuni*	30.43	0.72		[36]
gdhF + gdhR	*G. lamblia*	37.75	0.71	Excluded due to higher Cp value	[37]
P241F + P241R *	*G. lamblia*	34.34	1.60		[38]
COWP P702F + COWP P702R *	*C. parvum*	25.96	0.11		[38]
CRULib13F + CRULib13R	*C. parvum*	26.81	3.91	Excluded due to higher Cp value	[39]
ybbw 401 F + 611 R	*E. coli*	33.16	0.74	Excluded due to unspecific product	[29]
uidA_F + uidA_R *	*E. coli*	33.14	0.47		[29]

**Table 2:** Sensitivity and efficiency of the qPCR against individual pathogens. Efficiency %—amplification efficiency. Limit of quantification (LoQ) and limit of detection (LoD) determined via serial dilution (Supplementary Table S4). * Expressed as cells or genome copies/PCR reaction.

Organisms	R^2^	Efficiency %	Limit of Quantification (LoQ) *	Limit of Detection (LoD) *
*C. jejuni*	0.9944	89.76	2	2
*C. parvum*	0.9985	96.45	4	4
*G. lamblia*	0.9967	86.50	12	5
*E. coli*	0.9835	89.04	19	2

**Table 3:** Performance of the qPCR primers in a mixed-pathogen environment. Pathogen mixture contained *C. jejuni*, *C. parvum*, *G. lamblia*, and *E. coli* in a 10 mL culture. Relative quantification efficiency (rQE%) determined used single pathogen cell count as benchmark (REF) and calculated as: rQE% = QE in mixed-pathogen sample/QE in single-pathogen sample × 100%. Primers against *E. coli* were not tested but have been included in the 100 mL-scale test (Table 6). See Section 3.5 quantification and statistical analysis for full details.

Samples	Primers	MeanCp	Estimated Cell Count/Reaction	Relative Quantification Efficiency (rQE %)
*C. jejuni* only	HipO-F + HipO-R	31.58	59.4	REF
*C. parvum* only	COWP 702F + COWP 702R	28.97	302.8	REF
*G. lamblia* only	P241F + P241R	33.46	20.0	REF
*E. coli* only	uidA_F + uidA_R	32.76	29.9	REF
Pathogen mixture	HipO-F + HipO-R	34.22	52.6	89%
Pathogen mixture	COWP 702F + COWP 702R	31.51	248.0	82%
Pathogen mixture	P241F + P241R	35.68	23.92	119%
Pathogen mixture	uidA_F + uidA_R	-	-	Not tested

**Table 4:** Evaluation of the effectiveness of tap water pre-treatment strategies for the removal of inhibitors in 10 mL samples. TW—tap water in. Treatment process of each sample detailed in corresponding column. * Treatment method resulted in filter blockage.

Scheme	Quantification Efficiency (QE)	Treatment Process	Main Application/Target(s) for Removal
*Pre-treatment-tap water samples treated prior to cell spiking*			
MQ	100%	Milli-Q H_2_O	Control: non-inhibiting
TW	40–60%	Untreated tap water	Control: inhibited, non-treated
PF-TW	60–80%	Pre-filtered tap water (0.2 µm polycarbonate)	Suspended particulates
ZL-TW	79%	TW pre-treated with zeolite, 2 cm bed height, 250–300 mesh	Heavy metal [54,55], humic acid, anions, organic matters, ammonia [56]
AZL-TW	44%	TW pre-filtered by Avoca zeolite, 150–250 mesh	
AC-TW	12% *	TW pre-treated by activated carbon (2 mL AC in 10 mL TW)	Trace organics, ammonia [57] Humic substances, organic matters, phenols, pesticides [58]
AC/MP-TW	103% *	TW pre-treated with milk protein-coated activated carbon	
Si8.1-TW	66%	TW pre-treated with silica pellets size 800 µm (1 cm bed height)	Suspended solids in water [59] Heavy metal, oil, organic pollutants [60]
Si5.2-TW	102%	TW pre-treated with silica pellets size 500 µm (2 cm bed height)	
Si50-TW	13%	TW pre-treated with silica sand 50 µm, acid washed	
SiSieve-TW	9%	TW pre-treated with silica molecular sieve type X13 (8 × 12 mesh)	
SPC18-TW	66%	TW pre-treated with SEP-PAK C18 column	Desalting, trace organics
ATSi-TW	59%	TW pre-treated with Alltech silica column	
ATDiol-TW	74%	TW pre-treated with Alltech Diol column	
StratSi-TW	53%	TW pre-treated with StrataX silica column	
CX-TW	12%	TW pre-treated with Chelex-100	Chelating functional group that binds and removes polyvalent metal ions
G-TW	101%	TW centrifuged before filter capture	Suspended particulates

**Table 5:** Evaluation of the effectiveness of treatment strategies in 10 mL of spiked tap water samples. TWC—tap water with spiked *C. jejuni* cells.

Sample	Quantification Efficiency (QE)	Treatment Process	Comment
*Post-treatment-tap water treated after cell spiking*			
TWC-ZL	1%	TWC treated with zeolite, 250–300 mesh	
TWC-Si5.2	30–100%	TWC treated with silica pellets, pellets size 500 µm (2 cm bed height)	Blockage, requires several filters
TWC-CX	26%	TWC treated with Chelex-100	
TWC-AC/MP	61%	TWC treated with milk protein-coated activated carbon	Blockage due to milk protein and AC dust
*Filter treatment-filters treated after filter capture process*			
TWC-HCl	>96%	Filter washed with 4 mL of 0.5 M HCl after capture process	

**Table 6:** Quantification efficiency (QE) of individual pathogens in a 100 mL mix-pathogen tap water sample treated with 20% phosphoric acid and rinsed with 3 mL of MilliQ-H_2_O. QE% values exceeding 100% likely due to Cp fluctuations in qPCR.

Target	Quantification Efficiency (%)	STD
*C. jejuni*	87–92	9%
*C. parvum*	103–114	6%
*G. lamblia*	86–97	3%
*E. coli*	99	4%

## Data Availability

Not applicable.

## Supplementary Materials

The following are available online at www.mdpi.com/2076-2607/9/11/2367/s1, Table S1: List of primers used for quantification PCR against *C. jejuni*, *C. parvum*, *G. lamblia* and *E. coli*, Table S2: Optimal concentrations for primer combinations as determined by titration, Table S3: Final concentration of primer pairs used for primer titration experiment, Table S4: Sensitivity of qPCR against the four pathogens, Table S5: Results of water analysis—inorganics and results of water analysis—chlorine, fluoride and CaCO3, Table S6: Overall quantification or recovery efficiency of molecular-based pathogen quantification workflows.
