# Supplementary material for: Universal Enzyme-Based Field Workflow for Rapid and Sensitive Quantification of Water Pathogens
Source: Microorganisms. 2021 Nov 16;9(11):2367. doi: 10.3390/microorganisms9112367 (PMC8618791; doi:10.3390/microorganisms9112367)
Supplement: Supplementary file 1 [file microorganisms-09-02367-s001.zip › microorganisms-1448322-supplementary.pdf]

*microorganisms*

Supplementary Material

# Universal Enzyme-Based Field Workflow for Rapid and Sensitive Quantification of Water Pathogens

**Angela Sun [1], Jo-Ann L. Stanton [2,*] Peter L. Bergquist [1,3,] and Anwar Sunna [1,4]**

1 Department of Molecular Sciences, Macquarie University, Sydney, NSW 2109, Australia; angela.sun@mq.edu.au(A.S1) ; peter.bergquist@mq.edu.au (PL.B); anwar.sunna@mq.edu.au (A.S2)
2 Department of Anatomy, School of Biomedical Sciences, University of Otago, PO Box 56, Dunedin 9054, New Zealand; jo.stanton@otago.ac.nz
3 Department of Molecular Medicine & Pathology, University of Auckland, Auckland, New Zealand
4 Biomolecular Discovery Research Centre, Macquarie University, Sydney, NSW 2109, Australia

* Correspondence: jo.stanton@otago.ac.nz (J.S)

**Supplementary Table S1.** List of primers used for quantification PCR against *C. jejuni*, *C. parvum*, *G. lamblia* and *E. coli*. Primer sets marked with asterisks (*) were selected for further optimisation and quantification.

| Organism | Primer name | Target gene | Amplicon size (bp) | Sequence | Reference |
|---|---|---|---|---|---|
| *Campylobacter jejuni* | *CJ HipO-F<br>*CJ HipO-R | *HipO* | 100 | 5' TGCTAGTGAGGTTGCAAAAGAATT 3'<br>5' TCATTTCGCAAAAAAATCCAAA 3' | [1] |
| *Cryptosporidium parvum* | *COWP P702F<br>*COWP P702R | *COWP* | 151 | 5′ CAAATTGATACCGTTTGTCCTTCTG 3′<br>5′ GGCATGTCGATTCTAATTCAGCT 3′ | [2] |
| | CRULib13F<br>CRULib13R | *Lib13* | 166 | 5′ TCCTTGAAATGAATATTTGTGACTCG 3′<br>5′ TTAATGTGGTAGTTGCGGTTGAAC 3′ | [3] |
| *Giardia lamblia* | gdhF<br>gdhR | *gdh* | 261 | 5′ GGGCAAGTCCGACAACGA 3′<br>5′ GCACATCTCCTCCAGGAAGTAGAC 3′ | [4] |
| | *P241F<br>*P241R | *β-Giardin* | 75 | 5′CATCCGCGAGGAGGTCAA 3′<br>5′GCAGCCATGGTGTCGATCT 3 | [2] |
| *Escherichia coli* | 401 F<br>611 R | *ybbW* | 211 | 5′ TGATTGGCAAAATCTGGCCG 3′<br>5′ GAAATCGCCCAAATCGCCAT 3′ | [5] |
| | *uidA_F<br>*uidA_R | *uidA* | 70 | 5′ CGGAAGCAACGCGTAAACTC 3′<br>5′ TGAGCGTCGCAGAACATTACA 3′ | [5] |

**Supplementary Table S2** Optimal concentrations for primer combinations as determined by titration. Mean and STD Cp calculated from triplicate reactions. Highlighted cells indicate best performing primer set against each pathogen that were used for all subsequent experiments. Abbreviations: F/R – forward/reverse primers. 100-500 nm -final concentration of 100-500 nM per reaction. Highlighted cells – best performing primer concentrations.

| | ***C. jejuni*** | | ***C. parvum*** | | ***G. lamblia*** | | ***E. coli*** | |
|---|---|---|---|---|---|---|---|---|
| Primers used | HipO-F + HipO-R | | COWP 702F + COWP 702R | | P241F + P241R | | uidA_F + uidA_R | |
| Primer concentrations | MeanCp | STD Cp | Mean Cp | STD Cp | MeanCp | STD Cp | MeanCp | STD Cp |
| F100 nm - R100 nm | 27.53 | 0.06 | 24.78 | 0.03 | 29.33 | 0.26 | 27.48 | 0.19 |
| F100 nm - R250 nm | 27.47 | 0.02 | 24.84 | 0.03 | 29.59 | 0.27 | 27.01 | 0.12 |
| F100 nm - R500 nm | 27.48 | 0.03 | 24.77 | 0.10 | 29.56 | 0.25 | 26.06 | 0.10 |
| F250 nm - R100 nm | 26.90 | 0.03 | 23.89 | 0.02 | 27.91 | 0.19 | 26.34 | 0.04 |
| F250 nm - R250 nm | 27.12 | 0.05 | 24.08 | 0.02 | 28.35 | 0.09 | 25.89 | 0.02 |
| F250 nm - R500 nm | 27.16 | 0.04 | 24.03 | 0.04 | 28.54 | 0.15 | 25.93 | 0.04 |
| F500 nm - R100 nm | 26.88 | 0.04 | 23.81 | 0.02 | 27.18 | 0.06 | 26.06 | 0.13 |
| F500 nm - R250 nm | 27.07 | 0.06 | 24.00 | 0.03 | 27.65 | 0.11 | 25.85 | 0.03 |
| F500 nm - R500 nm | 26.98 | 0.04 | 24.03 | 0.03 | 27.81 | 0.30 | 25.89 | 0.02 |

**Supplementary Table S3.** Final concentration of primer pairs used for primer titration experiment.

| | | **Forward (F) primer** | | |
|---|---|---|---|---|
| | | 100 nM | 250 nM | 500 nM |
| **Reverse (R) primer** | 100 nM | F100-R100 | F250-R100 | F500-R100 |
| | 250 nM | F100-R250 | F250-R250 | F500-R250 |
| | 500 nM | F100-R500 | F250-R500 | F500-R500 |

**Supplementary Table S4**. Sensitivity of qPCR against the four pathogens. Abbreviation 1E-1 to 1E-6 – serial dilutions of pathogen templates, NTC – No template control. CV%- coefficient of variation. LoQ – limit of quantification. LoD – limit of detection. NA – no amplification. UA – unspecific amplification based on melt curve analysis. Linear regression line established with series of six ten-fold dilutions. LgY- log of cell numbers/genome copies, lgX- log of mean Cp.

| **Samples** | **Cp (mean)** | **Cp (SD)** | **Estimated genome copies/cell count (mean)** | **Estimated genome copies/cell count (SD)** | **CV (%)** | **Sensitivity** | **Linear regression line** |
|---|---|---|---|---|---|---|---|
| *C. jejuni* 1E-1 | 24.50 | 0.04 | 7107.12 | 209.51 | 3% | | lgY=(lgX-1.5937)/-0.0531 |
| *C. jejuni* 1E-2 | 27.58 | 0.05 | 766.25 | 25.46 | 3% | | |
| *C. jejuni* 1E-3 | 30.67 | 0.11 | 103.43 | 6.87 | 7% | | |
| *C. jejuni* 1E-4 | 33.45 | 0.32 | 20.17 | 3.30 | 16% | | |
| *C. jejuni* 1E-5 | 37.84 | 0.87 | 1.98 | 0.69 | 35% | LoQ, LoD | |
| *C. jejuni* NTC | *NA* | - | - | - | - | - | |
| *G. lamblia* 1E-1 | 29.34 | 0.30 | 326.55 | 63.04 | 19% | | lgY = (lgX-1.5849)/-0.0467 |
| *G. lamblia* 1E-2 | 30.26 | 0.22 | 169.31 | 24.02 | 14% | | |
| *G. lamblia* 1E-3 | 32.67 | 0.15 | 32.73 | 3.04 | 9% | | |
| *G. lamblia* 1E-4 | 34.28 | 0.50 | 11.71 | 3.14 | 27% | LoQ | |
| *G. lamblia* 1E-5 | 35.67 | 1.86 | 4.99 | 3.31 | 66% | LoD | |
| *G. lamblia* NTC | *UA* | - | - | - | - | - | |
| *C. parvum* 1E-1 | 19.14 | 0.05 | 79780.35 | 3457.10 | 4% | | lgY= (lgX-1.55)/-0.0547 |
| *C. parvum* 1E-2 | 21.98 | 0.01 | 6339.47 | 42.90 | 1% | | |
| *C. parvum* 1E-3 | 25.00 | 0.01 | 603.88 | 2.08 | 1% | | |
| *C. parvum* 1E-4 | 27.72 | 0.10 | 91.18 | 5.75 | 6% | | |
| *C. parvum* 1E-5 | 29.72 | 0.29 | 25.57 | 4.18 | 16% | | |
| *C. parvum* 1E-6 | 32.95 | 0.70 | 3.88 | 1.24 | 32% | LoD, LoQ | |
| *C. parvum* NTC | *NA* | - | - | - | - | - | |
| *E. coli* 1E-4 | 30.80 | 0.05 | 95.64 | 3.12 | 3% | | lgY = (lgX-1.6836)/-0.0481 |
| *E. coli* 1E-5 | 33.60 | 0.41 | 18.59 | 3.78 | 20% | LoQ | |
| *E. coli* 1E-6 | 38.03 | 1.61 | 1.80 | 0.98 | 54% | LoD | |
| *E. coli* NTC | *NA* | - | - | - | - | - | |

**Supplementary Table S5.1** Results of water analysis – inorganics.

Page 1 of 1

**Australian Government**
**National Measurement Institute**

## QUALITY ASSURANCE REPORT

**Client:** **MACQUARIE UNIVERSITY**

**NMI QA Report No:** MACQ20/201016 T1

**Sample Matrix:** **Water**

| Analyte | Method | LOR | Blank | Duplicates | | | Recoveries | |
|---|---|---|---|---|---|---|---|---|
| | | | | Sample | Duplicate | RPD | LCS | Matrix Spike |
| | | ug/L | ug/L | ug/L | ug/L | % | % | % |
| **Inorganics Section** | | | | N20/024380 | | | | N20/024380 |
| Arsenic | NT2.47 | 1 | <1 | <1 | <1 | NA | 90 | 102 |
| Cadmium | NT2.47 | 0.1 | <0.1 | <0.1 | <0.1 | NA | 94 | 96 |
| Chromium | NT2.47 | 1 | <1 | <1 | <1 | NA | 93 | 105 |
| Copper | NT2.47 | 1 | <1 | 160 | 160 | 2 | 96 | 97 |
| Lead | NT2.47 | 1 | <1 | <1 | <1 | NA | 101 | 98 |
| Mercury | NT2.47 | 0.1 | <0.1 | <0.1 | <0.1 | NA | 95 | 95 |
| Nickel | NT2.47 | 1 | <1 | 2.4 | 2.5 | NA | 96 | 104 |
| Zinc | NT2.47 | 1 | <1 | 32 | 33 | 0 | 85 | 98 |
| Calcium Total- **ppm** | NT2.47 | 0.005 | <0.005 | 7.2 | 7.3 | 1 | 98 | 100 |
| Magnesium Total-**ppm** | NT2.47 | 0.005 | <0.005 | 4.6 | 4.6 | 0 | 99 | 99 |
| PotassiumTotal-**ppm** | NT2.47 | 0.05 | <0.05 | 1.7 | 1.7 | 0 | 94 | 98 |
| Sodium Total-**ppm** | NT2.47 | 0.05 | <0.05 | 12 | 12 | 0 | 98 | 100 |

Filename = K:\Inorganics\Quality System\QA Reports\TE\QAR2020\Water\

Legend:
Acceptable recovery is 75-120%.
Acceptable RPDs on duplicates is 44% at concentrations >5 times LOR. Greater RPD may be expected at <5 times LOR.
LOR = Limit Of Reporting
ND = Not Determined
RPD = Relative Percent Difference
NA = Not Applicable
LCS = Laboratory Control Sample.
#: Spike level is less than 50% of the sample's concentration, hence the recovery data cannot be reported.

Comments:
Results greater than ten times LOR have been rounded to two significant figures.
This report shall not be reproduced except in full.

**Supplementary Table S5.2** Results of water analysis – chlorine, fluoride and $CaCO_3$.



**Australian Government**
**National Measurement Institute**

## QUALITY ASSURANCE REPORT

**Client:** Macquarie University Environment & Geography

**NMI QA Report No:** MACQ20/201016 QA **Sample Matrix:** water

| Analyte | Method | LOR | Blank | Duplicates | | | Recoveries | |
|---|---|---|---|---|---|---|---|---|
| | | | | Sample | Duplicate | RPD | Matrix spk | LCS |
| | | mg/L | mg/L | mg/L | mg/L | % | % | % |
| **Waters Section** | | | | N20/024379 | | | N20/024379 | |
| Chlorine - Total | NW_S16 | 0.05 | <0.05 | <0.05 | NA | NA | NA | 105 |
| Bicarbonate as CaCO3 | NW_B1 | 5 | <5 | <5 | NA | NA | NA | NA |
| Carbonate as CaCO3 | NW_B1 | 5 | <5 | 22 | NA | NA | NA | NA |
| Hydroxide as CaCO3 | NW_B1 | 5 | <5 | <5 | NA | NA | NA | NA |
| Alkalinity - Total as CaCO3 | NW_B1 | 5 | <5 | 20 | NA | NA | NA | 80 |
| Fluoride | NW_B3_B14 | 0.1 | <0.1 | 1.1 | NA | NA | NA | 111 |
| | | | | | | | | |

Filename = K:\Inorganics\WATER SECTION\Daily Work Wor

Legend
Acceptable recovery is 80-120%.
Acceptable RPDs on duplicates is 30% at > 5 times LOR. Greater RPD may be expected at < 5 LOR.
LOR = Limit Of Reporting
RPD = Relative Percent Difference
LCS = Laboratory Control Sample.
ND = Not Determined
NA = Not Applicable

Comments
This report shall not be reproduced except in full.
Results greater than ten times LOR have been rounded to two significant figures.

**Supplementary Table S6.** Overall quantification or recovery efficiency of molecular-based pathogen quantification workflows.

| Methods | Target organisms | Quantification/Recovery efficiency | | References |
|---|---|---|---|---|
| Filtration and qPCR | *Giardia spp.* and *Cryptosporidium spp.* | *Cryptosporidium* 70% | *Giardia* 84.9% | Al-Sabi *et al.* [6] |
| Immuno-separation (IMS)+ Fluorescent antibody (FA) + Filtration | *Giardia spp.* and *Cryptosporidium spp.* | *Cryptosporidium* FA 95% IMS+FA 92% Filtration+IMS+FA 18% | *Giardia* FA 94% IMS+FA 89% Filtration+IMS+FA 77% | Hu *et al.*[7] |
| Flow-cytometry, field deployment adaptations | *Giardia spp.* and *Cryptosporidium spp.* | *Cryptosporidium* 13% | *Giardia* 30% | Keserue *et al.* [8] |
| qPCR after DNA extraction | *Giardia spp.* and *Cryptosporidium spp.* | DNA extraction efficiency affected by inhibitors | | Guy *et al.*[2] |
| Microfluidics and qPCR | Multiple Food- and Waterborne Pathogens | qPCR efficiency 90-110% (30 pathogens) Recovery efficiency 22-27% (EHEC only) | | Ishii *et al.*[9] |
| Digital PCR and q PCR | *Campylobacter jejuni* | Incomplete recovery | | Papić *et al.* [10] |
| Direct qPCR, Integrated Cell Concentration and DNA Purification | *Campylobacter jejuni* | Subject to higher cell number, requires centrifugation | | Rudi *et al.* [11] |

## References

1. Granato, P.A.; Chen, L.; Holiday, I.; Rawling, R.A.; Novak-Weekley, S.M.; Quinlan, T.; Musser, K.A. Comparison of premier CAMPY enzyme immunoassay (EIA), ProSpecT *Campylobacter* EIA, and ImmunoCard STAT! CAMPY tests with culture for laboratory diagnosis of *Campylobacter* enteric infections. *J Clin Microbiol* **2010**, *48*, 4022-4027.
2. Guy, R.A.; Payment, P.; Krull, U.J.; Horgen, P.A. Real-time PCR for quantification of *Giardia* and *Cryptosporidium* in environmental water samples and sewage. *Appl. Environ. Microbiol.* **2003**, *69*, 5178-5185.
3. Hadfield, S.J.; Robinson, G.; Elwin, K.; Chalmers, R.M. Detection and differentiation of *Cryptosporidium* spp. in human clinical samples by use of real-time PCR. *J Clin Microbiol* **2011**, *49*, 918-924.
4. Yang, R.; Jacobson, C.; Gardner, G.; Carmichael, I.; Campbell, A.J.D.; Ryan, U. Development of a quantitative PCR (qPCR) for *Giardia* and analysis of the prevalence, cyst shedding and genotypes of *Giardia* present in sheep across four states in Australia. *Exp. Parasitol.* **2014**, *137*, 46-52.
5. Walker, D.I.; McQuillan, J.; Taiwo, M.; Parks, R.; Stenton, C.A.; Morgan, H.; Mowlem, M.C.; Lees, D.N. A highly specific *Escherichia coli* qPCR and its comparison with existing methods for environmental waters. *Water Res.* **2017**, *126*, 101-110.
6. Al-Sabi, M.N.; Gad, J.A.; Riber, U.; Kurtzhals, J.A.; Enemark, H.L. New filtration system for efficient recovery of waterborne *Cryptosporidium* oocysts and *Giardia* cysts. *J. Appl. Microbiol.* **2015**, *119*, 894-903.
7. Hu, J.; Feng, Y.; Ong, S.L.; Ng, W.J.; Song, L.; Tan, X.; Chu, X. Improvement of recoveries for the determination of protozoa *Cryptosporidium* and *Giardia* in water using method 1623. *J. Microbiol. Methods* **2004**, *58*, 321-325.
8. Keserue, H.-A.; Füchslin, H.P.; Wittwer, M.; Nguyen-Viet, H.; Nguyen, T.T.; Surinkul, N.; Koottatep, T.; Schürch, N.; Egli, T. Comparison of rapid methods for detection of *Giardia* spp. and *Cryptosporidium* spp. (Oo)cysts using transportable instrumentation in a field deployment. *Environ. Sci. Technol.* **2012**, *46*, 8952-8959.
9. Ishii, S.; Segawa, T.; Okabe, S. Simultaneous quantification of multiple food- and waterborne pathogens by use of microfluidic quantitative PCR. *Appl. Environ. Microbiol.* **2013**, *79*, 2891-2898.
10. Papić, B.; Pate, M.; Henigman, U.; Zajc, U.; Gruntar, I.; Biasizzo, M.; Ocepek, M.; Kušar, D. New approaches on quantification of campylobacter jejuni in poultry samples: the use of digital PCR and real-time PCR against the ISO standard plate count method. *Front. Microbiol.* **2017**, *8*, 331.
11. Rudi, K.; Høidal, H.K.; Katla, T.; Johansen, B.K.; Nordal, J.; Jakobsen, K.S. Direct real-time PCR quantification of *Campylobacter jejuni* in chicken fecal and cecal samples by integrated cell concentration and DNA purification. *Appl. Environ. Microbiol.* **2004**, *70*, 790-797.
